# Anabolic‐androgenic anabolic steroids as a probable risk factor for hepatoblastoma in infants

**DOI:** 10.1002/ccr3.7676

**Published:** 2023-07-10

**Authors:** Armen Malekiantaghi, Hosein Shabani‐Mirzaee, MohammadAli Ehsani, Kambiz Eftekhari

**Affiliations:** ^1^ Pediatric Gastroenterology, Pediatric Department Tehran University of Medical Sciences, Bahrami Children's Hospital Tehran Iran; ^2^ Pediatric Endocrinology and Metabolism, Pediatric department Tehran University of Medical Sciences, Bahrami Children's Hospital Tehran Iran; ^3^ Pediatric Hematology and Oncology, Department of Pediatric Hematology and Oncology Tehran University of Medical Sciences, Bahrami Children's Hospital Tehran Iran; ^4^ Pediatric Gastroenterology, Pediatric Gastroenterology and Hepatology Research Center, Pediatric Department Tehran University of Medical Sciences, Bahrami Children's Hospital Tehran Iran

**Keywords:** androgenic anabolic steroids, hepatoblastoma, infant, liver, paternal exposure

## Abstract

**Key Clinical Message:**

The etiology of hepatoblastoma (HB) is still unknown; several risk factors have been identified. The only risk factor for the development of HB in presented case was the child's father using anabolic androgenic steroids. It may be a risk factor for developing HB in their children.

**Abstract:**

HB is the most common primary liver cancer in children. Its etiology is still unclear. The patient's father's use of androgenic anabolic steroids could be a risk factor for developing HB in his child. A 14‐month‐old girl was hospitalized with intermittent fever, severe abdominal distention, and anorexia. On initial examination, she was cachectic and pale. There were two hemangioma‐like skin lesions in the back. Huge hepatomegaly was found and the ultrasound showed a hepatic hemangioma. The possibility of malignancy was considered due to the severe enlargement of the liver and the increased levels of the alpha‐fetoprotein. An abdominopelvic CT scan was performed and finally, the diagnosis of HB was confirmed by pathology. There was no history of congenital anomalies or risk factors for HB.

Also we did not find any risk factors in the mother's history either. The only positive finding in the father's history was the use of anabolic steroids for bodybuilding. Anabolic‐androgenic anabolic steroids may be one of the possible causes of HB in children.

## 1 INTRODUCTION

Liver tumors in children are rare and diverse.^1^ Primary liver tumors account for about 0.5%–2% of all pediatric cancers in the United States.^1^ More than two‐thirds of pediatric liver masses are malignant tumors, like hepatoblastoma (HB) in infancy and early childhood and hepatocellular carcinoma (HCC) in older children.^1^ These tumors are usually asymptomatic and are often characterized by palpable masses that are unfortunately unresectable at diagnosis.^1^ The average age of diagnosis of HB is 19 months. This tumor is most common in boys.^1^ Over the past two decades, the incidence of HB has increased and HCC has decreased.^2^ The reason for the increased prevalence of HB is the increased survival of premature infants (<1500 g),^3^ and the reduction in the prevalence of HCC is due to the increasing levels of hepatitis B vaccination.^4^ The etiology of HB, like many childhood cancers, is unclear.^5^ In some studies, several risk factors have been identified, including congenital anomalies such as Beckwith–Wiedemann syndrome, some trisomies (18 and 21), familial adenomatous polyps, pregnancy‐related factors such as oral contraceptive consumption, gestational hypertension, fetal alcohol syndrome, fertility with modern reproductive techniques, use of dyes and pigments by mothers,^6^ preeclampsia, poly or oligohydramnios, high pregnancy weight,^7^ parents' occupational exposure to heavy metal dust (iron, lead, tin, or copper),^6^ and parents who are smokers.^5^ In general, the most important risk factor associated with this tumor is low birth weight,^8^ especially very low birth weight (<1500 g).^5^ Anabolic androgenic anabolic steroids have been reported to cause hepatoma. These drugs are used in bodybuilding by young people. There have been reports of HCC in users of these drugs.^9^, ^10^ The incidence of cancer in children of mothers who use petroleum products, dyes and pigments is significantly higher. Childbirth and child health have been focused on the mother and the child, and less attention has been paid to the father's role. Exposure of the father to environmental and occupational factors before pregnancy is associated with bad consequences such as birth defects, malignancies, and other developmental concerns in his child.^11^ The relationship between childhood cancer and paternal risk factors is still controversial. However, the cause of most malignant liver tumors in children is still unknown.^1^

Our purpose in reporting this case is that the use of anabolic drugs by the father can be a probable risk factor for developing HB.

## 2 CASE PRESENTATION

**FIGURE 1:**
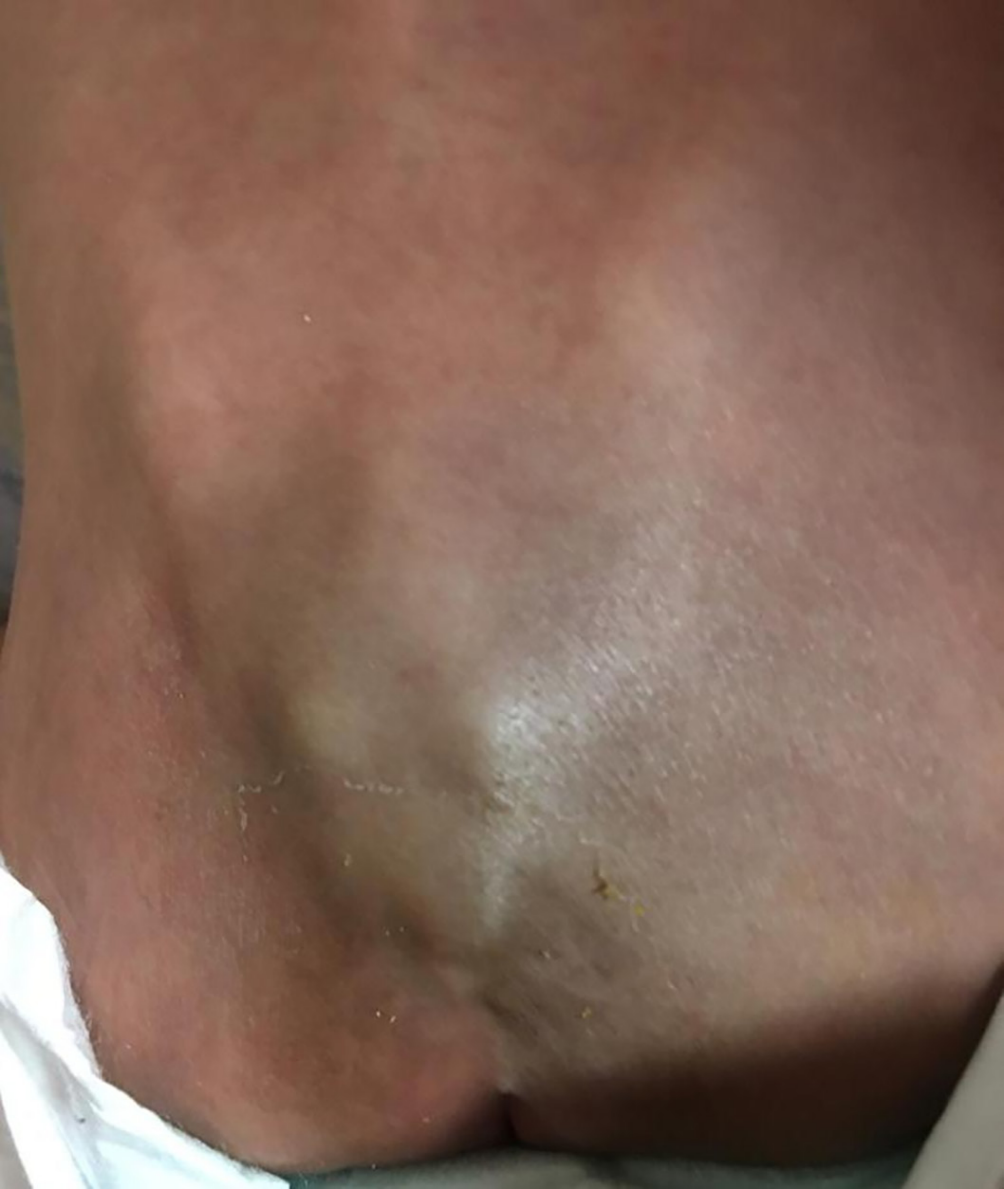
Blue‐purple patches of skin at the end of the lumbar spine and sacrum.

**FIGURE 2:**
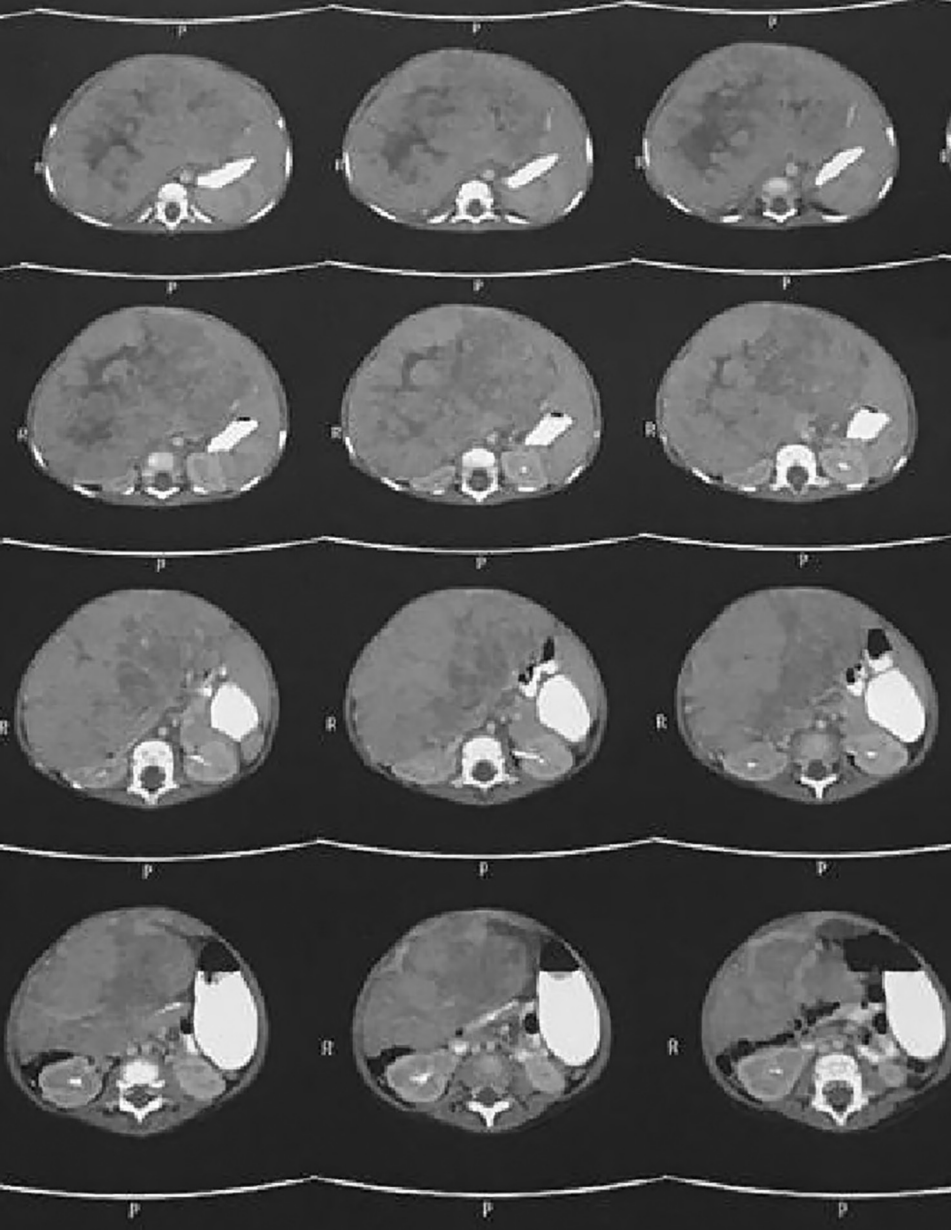
The liver CT scan.

A 14‐month‐old girl presented with intermittent fever and abdominal distention beginning 1 month before. She had anorexia and growth retardation during this time. She is the first child of non‐consanguineous parents, born with a birth weight of 3200 g and a height of 50 cm. Current weight was 7300 g (below the fifth percentile weight for age) and height 80 cm (above the 90th percentile height for age). In the initial examination, irritability following touch, severe cachexia, bilateral atrophy of the temporal muscles, severe pallor of the conjunctive, and symmetrical abdominal distension was evident. Cardiopulmonary examination was normal. In the abdomen, there was a huge hepatomegaly with a span of 18 cm that extended to the outskirts of the iliac crest. The spleen was not palpable. There was no peripheral lymphadenopathy. Two blue‐purple patches of skin similar to hemangiomas were seen in the back, one of them was in the cervical area and the other was at the end of the lumbar spine and sacrum (Figure 1). In early infancy, due to these skin lesions, she underwent abdominal ultrasound which reported a mass in the liver with the possibility of hemangioma. Initial lab tests, including liver function tests and inflammatory markers, were normal, but a very strong increase in the blood level of alpha‐fetoprotein (AFP > 20,000 ng/mL with a normal range less than 50 ng/mL) was the only abnormal finding. A liver malignancy was suspected due to the large size of the mass and tumor markers, so, an abdominopelvic CT scan was performed (Figure 2). CT scan showed extensive hepatomegaly, lobule contour and heterogeneity of liver tissue, which suggested malignant changes, especially HB. Open biopsy was done. The pathology report confirmed the diagnosis of HB. Chemotherapy was started with 5‐FU, vincristine, and cisplatin.

The child's mother became pregnant at the age of 36. She had a history of ectopic pregnancy and unilateral tubectomy. The mother was a housewife and had no occupational exposure to heavy metals. There was no history of smoking, use of alcohol, or any medication during pregnancy. Perinatal screenings were normal. The patient's father was 40 years old at that time. He also had no history of smoking or drinking alcohol but he had a history of taking anabolic steroid supplements (nandrolone decanoate at a dose of 100 mg every week for about 8 years) for bodybuilding.

## 3 DISCUSSION

Fifteen percent of all abdominal tumors in childhood are primary liver tumors, 66% of them malignant, and the most common is HB.^7^ This is a very rare cancer that originates in the liver. The most common symptoms are abdominal mass, distention, and pain.^1^ The liver is the main site of metabolism of many chemicals agents and as a result, they may become active carcinogens at this site.^8^ The carcinogenicity of carcinogens is greater in intrauterine, neonatal or hepatic regeneration period than in adulthood.^12^ The environmental factors involved in the development of HB are diverse, some of them include maternal alcohol abuse and fetal alcohol syndrome, the use of oral contraceptives in early pregnancy, smoking, and exposure to some heavy metals.^6^ Childbirth and child health have been focused on the mother and the child, and less attention has been paid to the role of father before conception, during pregnancy, and after birth.^11^ Abnormalities caused by fathers' use of drugs (therapeutic or recreational), occupational contact with chemicals, and ionizing radiation have been reported. These abnormalities include congenital abnormalities, spontaneous abortion, fetal death, low birth weight, increase in childhood cancers; abnormalities in the development, neurobehavioral, neuroendocrine, and effects in the offspring of the next generation.^13^

In this patient, the presence of cutaneous hemangiomas and ultrasound report initially raised the possibility of hepatic hemangiomas. Although the presence of cutaneous hemangiomas increases the risk of internal hemangiomas (such as hepatic), it is important to note that there are other accompanying symptoms. This includes the size of the mass, which is usually small in the hepatic hemangiomas. Therefore, the presence of larger tumors should raise suspicion of malignant masses. In this patient, with the presence of a mass and the subsequent manifestations, the diagnosis of hepatic hemangioma became less pronounced and additional examinations became necessary. The blood level of AFP is one of the best markers and high levels indicate liver malignancies, especially HB.^8^ This marker was very high in this child. On the other hand, all risk factors for HB, including genetic syndromes, congenital anomalies, maternal factors, occupational exposure, prematurity, low birth weight, neonatal problems, and the use of various drugs were absent. The only positive finding in the history was the long‐term use of anabolic steroids for bodybuilding in his father. The mechanism of liver damage secondary to anabolic steroids includes: the infiltration of inflammatory cells in the liver tissue and the activation of Kupffer cells, which leads to the production of inflammatory cytokines, increased oxidative stress, and mitochondrial degeneration in hepatocytes. And finally, it stimulates the intracellular steroid androgen receptors, which causes unregulated growth of hepatocytes.^14^

## 4 CONCLUSION

Father's use of anabolic androgenic anabolic steroids can be considered as one of the possible risk factors for HB in their children. Of course, this is a theory and needs further evaluation to be conclusive. However, warning against not taking such drugs and compounds is important for both mother and father.

## AUTHOR CONTRIBUTIONS

**Armen Malekiantaghi:** Conceptualization; data curation; investigation; methodology; project administration; software; supervision; visualization; writing – original draft; writing – review and editing. **Hosein Shabani‐Mirzaee:** Conceptualization; investigation; project administration; supervision; validation; visualization; writing – original draft; writing – review and editing. **MohammadAli Ehsani:** Conceptualization; investigation; methodology; software; supervision; writing – original draft; writing – review and editing. **Kambiz Eftekhari:** Conceptualization; data curation; investigation; methodology; project administration; supervision; validation; visualization; writing – original draft; writing – review and editing.

## FUNDING INFORMATION

No external funding was secured for this study.

## CONFLICT OF INTEREST STATEMENT

The authors had no conflict of interest.

## ETHICS STATEMENT

The written informed consent was taken from the child's parents to publish her case in the journal.

## CONSENT

Written informed consent was obtained from the patient to publish this report in accordance with the journal's patient consent policy.

## Data Availability

The dataset presented in the study is available on request from the corresponding author during submission or after publication. The data are not publicly available due to ethics.

## References

[1] López‐Terrada D , Finegold MJ . Liver Tumors. In: Kleinman RE , Sanderson IR , Goulet O‐J , et al., eds. Walker's Pediatric Gastrointestinal Disease. 6th ed. People's Medical Publishing House; 2018:3576‐3645.

[2] Hadzic N , Finegold MJ . Liver neoplasia in children. Clin Liver Dis. 2011;15(2):443‐462. vii‐x. 21689623 10.1016/j.cld.2011.03.011

[3] Turcotte LM , Georgieff MK , Ross JA , et al. Neonatal medical exposures and characteristics of low birth weight hepatoblastoma cases: a report from the Children's Oncology Group. Pediatr Blood Cancer. 2014;61(11):2018‐2023. 25044669 10.1002/pbc.25128 PMC4287257

[4] Chang MH . Hepatitis B virus and cancer prevention. Recent Results Cancer Res. 2011;188:75‐84. 21253790 10.1007/978-3-642-10858-7_6

[5] Spector LG , Birch J . The epidemiology of hepatoblastoma. Pediatr Blood Cancer. 2012;59(5):776‐779. doi:10.1002/pbc.24215 22692949

[6] Reynolds P , Urayama KY , Von Behren J , Feusner J . Birth characteristics and hepatoblastoma risk in young children. Cancer. 2004;100(5):1070‐1076. doi:10.1002/cncr.20061 14983504

[7] Pateva IB , Egler RA , Stearns DS . Hepatoblastoma in an 11‐year‐old: case report and a review of the literature. Medicine (Baltimore). 2017;96(2):e5858. doi:10.1097/MD.0000000000005858 28079820 PMC5266182

[8] Meyers RL , Maibach R , Hiyama E , et al. Risk‐stratified staging in paediatric hepatoblastoma: a unified analysis from the Children's Hepatic tumors International Collaboration. Lancet Oncol. 2017;18:122‐131. doi:10.1016/S1470-2045(16)30598-8 27884679 PMC5650231

[9] Hardt A , Stippel D , Odenthal M , Hölscher AH , Dienes HP , Drebber U . Development of hepatocellular carcinoma associated with anabolic androgenic steroid abuse in a young bodybuilder: a case report. Case Rep Pathol. 2012;2012:195607. doi:10.1155/2012/195607 22934212 PMC3420693

[10] Woodward C , Smith J , Acreman D , Kumar N . Hepatocellular carcinoma in body builders; an emerging rare but serious complication of androgenic anabolic steroid use. Ann Hepatobiliary Pancreat Surg. 2019;23(2):174‐177. doi:10.14701/ahbps.2019.23.2.174 31225420 PMC6558130

[11] Kothari A , Thayalan K , Dulhunty J , Callaway L . The forgotten father in obstetric medicine. Obstet Med. 2019;12(2):57‐65. doi:10.1177/1753495X18823479 31217809 PMC6560841

[12] Buckley JD , Sather H , Ruccione K , et al. A case‐control study of risk factors for hepatoblastoma. A report from the Childrens Cancer Study Group. Cancer. 1989;64(5):1169‐1176. doi:10.1002/1097-0142 2547509

[13] Friedler G . Paternal exposures: impact on reproductive and developmental outcome, an overview. Pharmacology Biochemistry and Behavior. 1996;55(4):691‐700. doi:10.1016/S0091-3057(96)00286-9 8981601

[14] Petrovic A , Vukadin S , Sikora R , et al. Anabolic androgenic steroid‐induced liver injury: an update. World J Gastroenterol. 2022;28(26):3071‐3080. doi:10.3748/wjg.v28.i26.3071 36051334 PMC9331524

